# The factors that influence postoperative stability of the dental implants in posterior edentulous maxilla

**DOI:** 10.1186/s40902-016-0100-2

**Published:** 2017-01-05

**Authors:** Yun-Ho Kim, Na-Rae Choi, Yong-Deok Kim

**Affiliations:** Department of Oral and Maxillofacial Surgery, School of Dentistry, Pusan National University, Yangsan, Republic of Korea

**Keywords:** Implant, Posterior maxilla, Survival rate, Impact factor

## Abstract

**Background:**

All clinicians are aware of the difficulty of installing a dental implant in posterior maxilla because of proximate position of maxillary sinus, insufficient bone width, and lower bone density. This study is to examine which factors will make the implantation in the posterior maxilla more difficult, and which factors will affect the postoperative implant stability in this region.

**Methods:**

Five hundred seventy-three fixtures on the maxilla posterior were included for this study from all the patients who underwent an installation of the dental implant fixture from January 2010 to December 2014 at the Department of Oral and Maxillofacial Surgery in Pusan National University Dental Hospital (Yangsan, Korea). The postoperative implant stability quotient (ISQ) value, fixture diameter and length, presence of either bone graft or sinus lift, and graft material were included in the reviewed factors. The width and height of the bone bed was assessed via preoperative cone beam CT image analysis. The postoperative ISQ value was taken just before loading by using the OsstellTM mentor® (Integration Diagnostics AB, Gothenburg, Sweden). The *t* test and ANOVA methods were used in the statistical analysis of the data.

**Results:**

Mean ISQ of all the included data was 79.22. Higher initial bone height, larger fixture diameter, and longer fixture length were factors that influence the implant stability on the posterior edentulous maxilla. On the other hand, the initial bone width, bone graft and sinus elevation procedure, graft material, and approach method for sinus elevation showed no significant impact associated with the implant stability on the posterior edentulous maxilla.

**Conclusions:**

It is recommended to install the fixtures accurately in a larger diameter and longer length by performing bone graft and sinus elevation.

## Background

All clinicians are aware of the difficulty of installing a dental implant in the posterior maxilla. The implantation on the maxillary molar area is in a proximate position with the maxillary sinus, so there is a risk of perforation during the implantation of the dental implant fixture, and the maxilla proximate maxillary sinus usually has insufficient bone width for implantation. Furthermore, the maxilla has a lower bone density than that of the mandible, so it will need more time to ascertain the osseointegration of the implant fixture [1–4].

Unfortunately, these barriers have not yet been entirely resolved. In this study, we are planning to examine which factors will make the implantation in the posterior maxilla more difficult, and which factors will affect the postoperative implant stability in this region. In addition, we are going to provide relevant information by comparing our results with that of the previous reports.

## Methods

### Case selection

All the patients in this study underwent an installation of the dental implant fixture from January 2010 to December 2014 at the Department of Oral and Maxillofacial Surgery in Pusan National University Dental Hospital (Yangsan, Korea).

Among these patients, the cases with the implantation of one or more fixtures on the posterior edentulous maxilla were chosen. The 573 fixtures on the maxilla posterior were included for this study from a total of 1637 fixtures.

All of the fixtures, namely, US II® (Osstem implant, Seoul, Korea), Solar® (Shinhung, Seoul, Korea), and SLActive® (Straumann, Basel, Swiss) underwent surface treatment processes, such as sandblasting, large grit, and acid etching. All of the surgeries have been performed by a single surgeon.

This study was exempted by the IRB review in Pusan National University Dental Hospital.

### Methods

First, all of the charts of the patients were reviewed. The postoperative implant stability quotient (ISQ) value, fixture diameter and length, presence of either bone graft or sinus lift, and graft material were included in the reviewed factors. The width and height of the bone bed were assessed via preoperative cone beam CT image analysis. In case bone graft or sinus lift was performed, only the initial pre-graft bone measurement was used in this study and not the post-graft state. The postoperative ISQ value was taken just before loading by using the OsstellTM mentor® (Integration Diagnostics AB, Gothenburg, Sweden). The *t* test and ANOVA methods were used in the statistical analysis of the data. Analyses were performed using Microsoft Excel 2010® (Microsoft, Redmond, USA).

The exclusion criteria included the cases with follow-up loss and loss of ISQ value taking. In the case of bone graft or sinus lift accompaniment, the patients without having taken either a preoperative or postoperative CBCT image were also excluded.

This study excluded the 1st premolar case, which is distant from the maxillary sinus and usually with sufficient bone volume for implantation, as it might be contradictory to the purpose of this study.

## Results

Five hundred seventy-three fixtures were collected, but among them, 91 fixtures were excluded according to exclusion criteria. And 1 fixture failed before loading. Overall survival rate was 99.8% by the moment just before loading (481/482).

Table 1 summarized the result of the implant stability based on sex and age. The male subjects showed a higher ISQ value with a statistical significance (*P* = 0.03). By age, the group with subjects older than 80 years old showed a much higher ISQ with a statistical significance (*P* = 0.02).

**Table 1:** Postoperative ISQ value by sex & age. *T* test & ANOVA were performed using Microsoft Excel 2010®

Age	<29	30 ~ 39	40 ~ 49	50 ~ 59	60 ~ 69	70 ~ 79	>80	Total	ISQ by sex (*P* = 0.03)
Sex									
Male	3	16	63	102	86	23	3	296	79.78 ± 6.82
Female	3	10	35	83	46	7	1	185	78.34 ± 7.63
Total	6	26	98	185	132	30	4	481	
ISQ by age (*P* = 0.02)	79.33 ± 3.44	76.34 ± 9.15	78.65 ± 9.17	78.98 ± 6.52	80.4 ± 5.82	78.6 ± 6.42	88.5 ± 5.45		Mean ISQ 79.22 ± 7.16

The characteristics of the underlying bone and implant fixtures were also inspected (Table 2). The ISQ value of the fixtures with a diameter equal to 5 mm or wider than 5 mm was significantly higher than that of the smaller diameter group (*P* = 0.003). Meanwhile, the longer length fixture group showed a significantly higher postoperative ISQ value (*P* = 0.002).

**Table 2:** Postoperative ISQ value by fixture diameter and length. *T* test was performed using Microsoft Excel 2010®

	Fixture diameter		Fixture length	
	<5 mm	≥5 mm	<11 mm	≥11 mm
*n*	255	218	258	214
ISQ	78.33 ± 7.12	80.29 ± 7.14	78.33 ± 7.67	80.35 ± 6.41
	(*P* = 0.003)		(*P* = 0.002)	

The volume of the underlying bone bed was measured in terms of width and height (Table 3). The postoperative stability showed no significant difference according to fixture diameter (*P* = 0.50). In contrast, a longer initial bone height showed a significantly increased postoperative ISQ value (*P* = 0.035).

**Table 3:** Postoperative ISQ value by initial bone width and height. *T* test and ANOVA were performed using Microsoft Excel 2010®

	Bone width		Bone height			
	<6 mm	≥6 mm	<4 mm	4 ~ 8 mm	8 ~ 12 mm	>12 mm
*n*	128	295	158	141	90	41
ISQ	79.72 ± 7.20	79.20 ± 7.32	78.25 ± 8.46	79.43 ± 6.39	80.37 ± 6.21	81.37 ± 6.55
	(*P* = 0.50)		(*P* = 0.035)			

In addition, the postoperative ISQ value was measured according to diverse criteria, such as bone graft or graft materials, sinus elevation, and surgical techniques. The results are summarized in Table 4. There was no significant factor associated with postoperative stability.

**Table 4:** Postoperative ISQ value based on whether bone graft was accompanied, by graft material, whether sinus lift was accompanied, between approach techniques. *T* test & ANOVA were performed using Microsoft Excel 2010®

		*n*	ISQ
Whether bone graft performed (*P* = 0.42)	With bone graft	354	79.08 ± 7.37
	Without bone graft	125	79.68 ± 6.57
By graft material (*P* = 0.52)	Autogenous	144	79.37 ± 7.19
	Xenograft	177	78.73 ± 7.28
	Mixture (autogenous + xenograft)	30	80.20 ± 8.51
Whether sinus lift performed (*P* = 0.14)	With sinus lift	301	78.87 ± 7.55
	Without sinus lift	174	79.84 ± 6.46
By approach technique of sinus lift (*P* = 0.16)	Lateral approach	263	78.70 ± 7.86
	Crestal approach	24	80.13 ± 4.26

Among 481 fixtures, there was no record of fixture diameter in 8 fixtures, and there was no record of fixture length in 9 fixtures. Fifty-eight fixtures had no record of bone width, 51 fixtures had no record of bone height, and 2 fixtures had no information about bone graft.

It was unknown whether sinus lift was performed in 6 fixtures. And, there was no record of approach technique in 14 of 301 fixtures in augmented sinus.

## Discussion

This study adopted the ISQ to estimate and evaluate the postoperative stability of the dental implant. The postoperative ISQ value was taken just before loading by using the OsstellTM mentor® (Integration Diagnostics AB, Gothenburg, Sweden).

In order to evaluate implant stability in the past, an invasive procedure was required, such as histologic examination or torque removal [5]. After that, the ISQ has been proposed as one of the non-invasive methods for implant stability assessment. This method with resonance frequency analysis (RFA) technology measures the amount of variation of tissue stiffness around the implant and gives relevant information about the bone-implant interface [6]. This is non-invasive, reproducible, and quick [5, 7]. It is also applicable at any point during treatment [6]. On the other hand, the ISQ is not so reliable in evaluating the mobile fixtures [7], and there is no definite guideline with regard to clinical use [6]. In spite of these limitations, the ISQ is currently considered as the best diagnostic tool due to its non-invasiveness and reliability for the evaluation of dental implant stability. A high ISQ value suggests subsequent successful osseointegration, while a low ISQ value implies marginal bone loss or the possibility of failure [6].

Based on the introduction, low bone density, presence of maxillary sinus in the adjacent anatomical position by later pneumatization, and insufficient bone bed create limitations in installing the implant fixture in the posterior maxilla [1–4]. As an alternative, short implant fixtures with 6-mm length or shorter than 6 mm have been suggested.

Lemos et al. [8] reported that short implants are similar to the standard implants in terms of failures, marginal bone resorption, or other complications. It is possible to create a predictable treatment plan with the short implant, especially in the case that needs an additional surgical procedure [8]. Pabst et al. [9] reported that using a short implant prevents the risk and costs associated with an augmentation procedure. Thoma et al. [10] reported that the short implant may be the preferred alternative because the patients are more satisfied with the lower cost and less surgical time of the short implant, rather than the long implant with more surgical interventions. Furthermore, there is no significant correlation between fixture length and implant failure in the posterior maxilla [9].

However, our findings slightly differed. As previously mentioned, researches have shown results that there are no clinical problems encountered with the short implant, but there are definite advantages to using the long implant. In this study, the higher postoperative stability results were confirmed by the longer length of the implant. Likewise, Winkler et al. [11] reported that the survival rate of the short implant was 66.7%, while the survival rate of the long implant was 96.4%.

On the other hand, some literature revealed that there are no definite differences in the survival rate between the short and long implants. In addition to the abovementioned study, the reported implants, which were shorter than 6-mm length, would be a good alternative treatment. Raviv et al. [12] demonstrated similar survival rates between short and long implants. Haas et al. [13] also reported that there are no significant influences with regard to the length of the implant fixture.

We also examined the differences in postoperative stability according to the fixture diameter. In the previous literature, the fixture diameter was not considered as a significant factor in a number of studies. Klein et al. [14] reported that there are no definite differences of survival rates between a narrow-diameter implant and a regular-diameter implant. Haas et al. [13] also reported that there are no significant influences with regard to the diameter of the implant fixture.

Many studies reported that diameter did not influence the long-term prognosis of the dental implants [15–18]. In particular, there were some existing studies, including this study, that have specifically examined the posterior edentulous maxilla area. One study showed that the narrow fixture diameter was not the cause of the implant failure in posterior maxilla [9]. In the other study, the fixture diameter was just secondary, and not the critical factor, while the other factors (e.g., oral hygiene) greatly affected the posterior maxilla [19]. In this study, the larger diameter fixtures showed a significantly higher ISQ value than the smaller diameter fixtures. Among the literature reviewed, one study reported that a wide-diameter implant can lead to bone resorption and atrophy of the periodontal tissue due to excessive occlusal force, which was contradictory to this study [20]. It is not a definitive result; therefore, when installing a wide-diameter implant, it needs careful management with a close follow-up evaluation.

Preoperative bone bed quantity is also thought to be influential on the postoperative implant stability, so we evaluated the preoperative bone bed status in terms of width and height. It was based on the initial bone volume, even if the augmentation procedure has been performed. It was to determine if the implants could be stabilized through bone grafts before implantation from small initial bone volume, and the fixtures were selected according to the diameter and length matching the sufficient bone volume after bone graft.

However, there are many studies regarding bone density, but it is difficult to find a similar previous study about the correlation of bone volume, quantity, and postoperative stability. It is deemed necessary to make further research. In one study about implants at augmented maxillary sinus, there was no significant correlation between the height of residual alveolar bone and the survival rate [21].

The postoperative ISQ value was also assessed, depending on whether the bone graft or sinus elevation procedure has been performed. In this study, both the bone graft and sinus elevation did not affect the postoperative stability. The postoperative ISQ value of the augmented maxillary sinus was not significantly different from the value at the native bone [9]. On the other hand, a previous research reported that there are three times more complications after a sinus augmentation has been performed [10]. In a similar study, there were more complications if the bone graft procedures were performed; however, the failure did not increase significantly [22]. It was apparent that the likelihood of complications would increase with more surgical procedures, but it is determined that there is no significant effect on postoperative stability if the operation is performed cautiously, based on a right surgical principle.

There was no significant difference found, according to the material used when a bone graft or sinus elevation was performed [23]. According to Bassi et al. [1], in the posterior maxilla, there was no definite abnormality found in the implant stability in the cases with sinus elevation only and without graft material. Markovic et al. [24] reported that when the fixture implantation was accompanied with sinus floor elevation in one stage, there was no definite advantage in the clinical success or implant stability, regardless of the presence or absence of the bone graft material. The result of this study and the result of the other literature showed that graft material does not have a significant impact on postoperative stability.

Based on sex and age, the male subjects showed a higher postoperative ISQ value than the female subjects in this study. It was statistically significant, but it might not be so reliable because it had a different result when compared to the existing studies. In general, it is known that the male subjects have a slightly lower implant survival rate due to several factors (e.g., smoking) [25–27], or there is no difference between the male and female subjects [28]. Based on age, there were some differences with the ISQ value among the age groups. However, it was difficult to find a definite correlation or tendency between age and implant stability. According to the Tukey test, which was conducted for a more detailed analysis, there was no definite relevance between age groups, except for the age groups between 30 years old and 80 years old. The result of this study was different from what was generally known. Moy et al. [28] reported that the implant failure rate of age group over 60 is higher than age group under 40 more than twice. Other researchers reported that no correlation was found between age and implant failure [27, 29]. The result of this study might be due to the problems regarding the unusual samples. Therefore, it is not reasonable to consider this result as absolutely reliable, but instead, it should only serve as reference.

Differences of ISQ value between cases of this study were not tremendous, but some of those were statistically significant, and these results will be meaningful and helpful for the clinicians. Although only single measured ISQ value could not represent the overall implant stability absolutely, ISQ is almost the only index that indicates the degree of osseointegration objectively, so this study adopted ISQ for the objective quantification of the implant stability. It needs further research about the method of objective evaluation of the osseointegration.

Overall survival rate was very high as 99.8%, although it was measured at the moment just before loading, after only several months from implantation. It is an encouraging result that suggests the possibility of high success of implant in posterior maxilla. Conrad et al. [30] also reported that high implant survival rate of 93.2% in posterior maxilla after 35.7 months.

## Conclusions

The initial bone height, fixture diameter, and fixture length are factors that influence the implant stability on the posterior edentulous maxilla. On the other hand, the initial bone width, bone graft and sinus elevation procedure, graft material, and approach method for sinus elevation do not affect the implant stability on the posterior edentulous maxilla.

Although postoperative stability is independent of the initial bone width, the implants on the posterior edentulous maxilla are more stable with a longer fixture length and a wider fixture diameter. Bone graft or sinus elevation procedure does not create a difference in stability, so it is recommended to install the fixtures accurately in a larger diameter and longer length by performing bone graft and sinus elevation.
